# Preparation, Characterization and Activity of a Peptide-Cellulosic Aerogel Protease Sensor from Cotton

**DOI:** 10.3390/s16111789

**Published:** 2016-10-26

**Authors:** J. Vincent Edwards, Krystal R. Fontenot, Nicolette T. Prevost, Nicole Pircher, Falk Liebner, Brian D. Condon

**Affiliations:** 1 Southern Regional Research Center, USDA, New Orleans, LA 70124, USA; krystal.fontenot@ars.usda.gov (K.R.F.); nicolette.prevost@ars.usda.gov (N.T.P.); brian.condon@ars.usda.gov (B.D.C.); 2 Division of Chemistry of Renewable Resources, University of Natural Resources and Life Sciences Vienna, Konrad-Lorenz-Straße 24, Tulln an der Donau A-3430, Austria; nicole.gk.pircher@gmail.com (N.P.); falk.liebner@boku.ac.at (F.L.)

**Keywords:** elastase peptide, cellulosic aerogels, human neutrophil elastase, biosensor, chronic wounds

## Abstract

Nanocellulosic aerogels (NA) provide a lightweight biocompatible material with structural properties, like interconnected high porosity and specific surface area, suitable for biosensor design. We report here the preparation, characterization and activity of peptide-nanocellulose aerogels (PepNA) made from unprocessed cotton and designed with protease detection activity. Low-density cellulosic aerogels were prepared from greige cotton by employing calcium thiocyanate octahydrate/lithium chloride as a direct cellulose dissolving medium. Subsequent casting, coagulation, solvent exchange and supercritical carbon dioxide drying afforded homogeneous cellulose II aerogels of fibrous morphology. The cotton-based aerogel had a porosity of 99% largely dominated by mesopores (2–50 nm) and an internal surface of 163 m^2^·g^−1^. A fluorescent tripeptide-substrate (succinyl-alanine-proline-alanine-4-amino-7-methyl-coumarin) was tethered to NA by (1) esterification of cellulose C6 surface hydroxyl groups with glycidyl-fluorenylmethyloxycarbonyl (FMOC), (2) deprotection and (3) coupling of the immobilized glycine with the tripeptide. Characterization of the NA and PepNA included techniques, such as elemental analysis, mass spectral analysis, attenuated total reflectance infrared imaging, nitrogen adsorption, scanning electron microscopy and bioactivity studies. The degree of substitution of the peptide analog attached to the anhydroglucose units of PepNA was 0.015. The findings from mass spectral analysis and attenuated total reflectance infrared imaging indicated that the peptide substrate was immobilized on to the surface of the NA. Nitrogen adsorption revealed a high specific surface area and a highly porous system, which supports the open porous structure observed from scanning electron microscopy images. Bioactivity studies of PepNA revealed a detection sensitivity of 0.13 units/milliliter for human neutrophil elastase, a diagnostic biomarker for inflammatory diseases. The physical properties of the aerogel are suitable for interfacing with an intelligent protease sequestrant wound dressing.

## 1. Introduction

Aerogels are solids that feature very low density, high specific surface area and consist of a coherent open-porous network of loosely-packed, bonded particles or fibers [1], which distinguishes them from other porous materials, like liquid foams, packed beds or open-porous metal foams. Their particular morphology in terms of high interconnected porosity (≤99.9 vol%) and large internal surface (≤1000 m²·g^−1^) render aerogels promising matrix materials for a variety of applications. Aerogels equipped with specific sensors can be used to detect changes in humidity [2], deformation strain [3,4], to report the presence of molecular compounds [5,6], organic vapors [7] or trigger a specific response to thermal [8] or photon [9,10] impact. Next to inorganic source materials (silica, alumina, clay and metal chalcogenides) [11,12], aerogels are increasingly prepared from organic polymers. Amongst them, biopolymer aerogels attract particular interest [13] and have been assessed as biocompatible scaffolds for tissue engineering [14,15] and biodegradable packaging foams [16]. Cellulose plays a central role in biopolymer aerogel research due to its abundance and accessibility next to intriguing material properties caused by macromolecular and supra-molecular specificities [12]. High hydrophilicity, purity and good biocompatibility are features that virtually invite using cellulose as biomaterials, e.g., materials in direct contact with living tissue as documented for bacterial cellulose. However, only little has been hitherto reported about the utilization of cellulosic aerogels as the host matrix for on biosensors capable of detecting large biomolecules or proteins. 

Point-of-care protease detection has received increased attention and has been applied clinically throughout the world to improve chronic wound treatment [17,18,19]. Moreover, peptide-based sensors are widely used in monitoring protease enzyme activity [20,21], cellular uptake of cryptophane [22] and caspase-3 activity [23,24]. They have a rich structure/function literature that can be utilized to design enzyme sensors and are central to a number of protease-based diseases and their therapy [8]. Furthermore, the growing use of three-dimensional (3D) structures, whether aerogels, microarrays, or supramolecular co-assembly polyrotaxane films, continues to rise as different interactions between small molecules-protein or protein-protein interactions are being evaluated to improve biosensing technologies [25,26]. The clinical application of peptide-based biosensor approaches to protease biomarker disease assessment in chronic wounds is growing, as well [19,27]. On the other hand, the combination of detection with wound dressing treatment of chronic wounds in a single dressing is not available at this time, and less attention has been given to the idea of incorporating a protease sensor in chronic wound dressings [28]. Thus, the ability to combine measurable protease detection with a dressing motif that removes proteases is a current goal of wound healing biomaterial design and wound diagnostic management [29]. 

Chronic wound fluid is comprised of cytokines, chemokines, growth factors, electrolytes and proteolytic enzymes [30,31,32]. Proteolytic enzymes, in particular matrix metalloproteases (MMP) and serine protease human neutrophil elastase (HNE), are responsible for proteolytic degradation of growth factors [19] and extracellular matrix (ECM) proteins. Acute wounds have normal levels of MMPs and HNE [33], which facilitates the clearance of cellular debris. However, chronic wounds have elevated levels of MMPs (0.1–0.2 U/mL) and HNE (0.02–0.1 U/mL) protease depending on the type of chronic wound (diabetic, venous pressure and arterial ulcers) [34]. These proteases at elevated concentrations are biomarkers for chronic wound treatment with protease sequestrant dressings [35,36,37]. Therefore, the use of nanocellulosic aerogels (NA) as the transducer surface attached to a fluorescent peptide substrate, such as succinyl-alanine-proline-alanine-4-amino-7-methyl-coumarin (Suc-Ala-Pro-Ala-AMC) or succinyl-alanine-alanine-proline-valine-4-amino-7-methyl-coumarin (Suc-Ala-Ala-Pro-Val-AMC) [38], which has selectivity for HNE, not only offers specificity, but also offers a way to detect HNE in chronic wound fluid. The fluorescent tripeptide-substrate (Suc-Ala-Pro-Ala-AMC) was tethered to a NA by (1) esterification of cellulose C6 surface hydroxyl groups with glycidyl-fluorenylmethyloxycarbonyl (FMOC), (2) deprotection and (3) coupling of the immobilized glycine with the tripeptide (peptide-nanocellulosic aerogel (PepNA)). Characterization of the NA and PepNA included techniques such as elemental analysis, mass spectral analysis, attenuated total reflectance infrared spectroscopy, nitrogen sorption at 77 K, scanning electron microscopy and bioactivity studies. The physical properties of the aerogel are suitable for interfacing with a protease sequestrant wound dressing.

## 2. Experimental Section

### 2.1. General

*N,N*-dimethylformamide (DMF) was purchased from VWR (Radnor, PA, USA)). Ethylcyanoglyoxylate-2-oxime (Oxyma Pure), diisopropylcarbodiimide (DIC), *N*,*N*-diisopropylethylamine (DIPEA), and 4-dimethylaminopyridine (DMAP) were purchased from Sigma Aldrich (St. Louis, MS, USA. Ammonium thiocyanate and calcium hydroxide and lithium chloride were purchased from Sigma Aldrich (Vienna, Austria). Fmoc-glycine-OH was purchased from Peptides International (Louisville, KY, USA). Absolute ethanol was purchased from Fisher Scientific (Vienna, Austria). Dimethyl sulfoxide (DMSO) was a product of Merck ( Vienna, Austria). All chemicals were used as received without further purification. Mechanically-cleaned greige cotton was cut with scissors into smaller fiber fragments prior to dissolution. Human neutrophil elastase (HNE) was purchased as a salt-free lyophilized solid from Athens Research and Technology (Athens, GA, USA) without further purification. The peptide n-succinyl-Ala-Pro-Ala-7-amino-4-methylcoumarin (Suc-Ala-Pro-Ala-AMC) was purchased from BACHEM (Torrance, CA, USA)) without further purification. Electrospray liquid chromatography mass spectrometry (ESI-LC/MS) data were collected on an Agilent 6520 QTOF LC-MS/MS instrument (Santa Clara, CA, USA)). 

### 2.2. Synthesis of Calcium Thiocyanate

Calcium thiocyanate was synthesized from calcium hydroxide and ammonium thiocyanate [39]. The water content of the obtained calcium thiocyanate octahydrate was determined by Karl-Fischer titration [40]. 

### 2.3. Dissolution of Greige Cotton Fiber Using Calcium Thiocyanate, Cellulose Coagulation, Solvent Exchange and Conversion of Alcogels to Aerogels

One wt% of chopped greige cotton fibers was dissolved in a mixture of calcium thiocyanate octahydrate and 2.5 wt% lithium chloride under continuous agitation at 140 °C for up to 3 h. Dissolution was observed by light microscopy (NOVEX Holland, B-Series, 200× magnification).

The viscous solutions were poured into cylindrical Teflon molds (height: 10 mm; diameter: 10 mm) and covered with ethanol. After completion of cellulose coagulation, the solidified bodies were thoroughly washed with deionized water (calcium thiocyanate octahydrate, transfer to water via 50 vol% ethanol) followed by absolute ethanol. Subsequently, the alcogels were placed into a 300-mL autoclave of a supercritical carbon dioxide (scCO_2_) drying unit equipped with a separator for carbon dioxide recycling (Separex, France). Extraction of ethanol was accomplished under constant flow of scCO_2_ (40 g/min) at 10 MPa and 40 °C (2–3 h). The system was then slowly and isothermally depressurized at a rate of <0.1 MPa/min. 

### 2.4. Esterification of Matrix 

To a 20-mL glass vial, Oxyma Pure (4.49 mmol), DIC (4.49 mmol) and DMAP (0.45 mmol) in DMF with Fmoc-Gly-OH (4.49 mmol) were added. The aerogel (4.49 mmol) was placed into a 50-mL centrifuge tube and the solution added and sonicated for 3 h at 25–30 °C. The esterified NA-Gly-Fmoc was washed thrice with DMF and allowed to remain in DMF for storage at ~4–8 °C. Prior to tethering of the fluorescent tripeptide, the glycine groups were deprotected by soaking NA-Gly-Fmoc with 20% piperidine/DMF solution and agitating for 3, 5 and 7 min while washing with DMF after each time period. The NA-Gly was then washed thrice with DMF followed by preparation for peptide coupling or washed thrice with DCM, air dried and stored for further use.

### 2.5. Immobilization of the Fluorescent Peptide 

To a 20-mL glass vial, Oxyma Pure (0.68 mmol), DIC (0.68 mmol) and DMAP (0.068 mmol) in DMF were added with the peptide substrate (0.052 mmol) in minimal DMF. The esterified transducer aerogel-Gly (0.67 mmol) was placed into a 50-mL centrifuge tube to which the solution was added. The 50-mL centrifuge tube was placed in an ultrasound bath while maintaining the temperature at 25–30 °C for 3 h and then placed in the refrigerator overnight. The peptide-nanocellulosic aerogel (PepNA) was purified by washing thrice with DMF and stored in DMF at ~4–8 °C until further use. Note: the biosensor will be defined as the tripeptide substrate immobilized on the NA-Gly.

Upon the completion of drying, the peptide was cleaved from 10 mg of each biosensor by adding a mixture of TFA/water/triisopropylsilane (95/2.5/2.5) to the biosensor for three hours. The solution for each biosensor was diluted with water (1:10) and submitted for ESI-LC/MS. The intact sequence of the peptide component cleaved from the biosensor was confirmed via its molecular weight. 

### 2.6. Elemental Analysis

The NA and PepNA samples were submitted to Midwest Microlabs for carbon (C), hydrogen (H) and nitrogen (N) analysis. Nitrogen content calculations for the nitrogen mass fraction of the tripeptide (net nitrogen %), total nitrogen content of PepNA excluding the contribution of glycine (nitrogen mass, μg), total tripeptide content of PepNA (peptide mass, μg), total tripeptide content of PepNA (peptide, %) and peptide mass fractions (μg/mg) were performed in Microsoft Excel 2007 using Equations (1)–(5). The net nitrogen percent is equal to the nitrogen percent of the peptide minus the nitrogen percent of glycine (Equation (1)). The nitrogen mass is calculated by multiplying the weight of the biosensor in mg by the nitrogen percent divided by 100 and multiplied by 1000 (Equation (2)). The peptide mass determines the amount of peptide on the surface of the biosensor by multiplying the nitrogen mass by the nitrogen factor of 6.25 [41] (Equation (3)). The peptide percentage is calculated by subtracting the nitrogen percent of glycine from the nitrogen percent of the peptide and multiplying that value by the nitrogen factor (6.25) [42] (Equation (4)). The amount of peptide per mg of support was achieved by dividing the peptide mass by the weight of the biosensor (Equation (5)).

(1) Net nitrogen %=nitrogen % of peptide−nitrogen % of glycine

(2) $$Nitrogen\;mass\;\left(\mu g\right)=weight\;of\;biosensor*\left(\frac{net\;nitrogen\;\%}{100}\right)*1000$$

(3) Peptide mass (μg)=Nitrogen mass*6.25

(4) Peptide %=(nitrogen % of peptide−nitrogen % of glycine)*6.25

(5) $$Peptide\;\left(\mu g/mg\right)=\frac{peptide\;mass}{weight\;of\;biosensor}$$

### 2.7. Degree of Substitution

The degree of substitution (D.S.) of the cellulose chain is the number of substituent groups attached per anhydroglucose repeating unit (AGU) [43]. The D.S. of the biosensors was calculated using Equation (6 [44], where PC is the percentage of nitrogen as determined by elemental analysis, MW_AGU_ is the molecular weight of one cellulose unit, MW_N_ is the molecular weight of one nitrogen atom, N represents the number of nitrogens and MW_PepNA_ is the molecular weight of the peptide including the glycine linker.

D.S. = PC(MW_AGU_)/[(MW_N_)(N)(100) − (MW_PepNA_)] (6)

### 2.8. Extraction of Pectin from PepNA and Mass Spectral Analysis

To a 50-mL beaker, 0.035 g of the NA and 20 mL of hexane were added and allowed to soak for two hours. The solution was sonicated for one minute every 30 min. After 2 h, the supernatant was isolated, concentrated and diluted 1:10 with water/acetonitrile (1/1). The samples were submitted to Louisiana State University Mass Spectral Facility for matrix-assisted laser desorption ionization time of flight mass spectrometry (MALD-TOF-MS); mass spectra were recorded on a Bruker UltrafleXtreme MALDI-TOF/TOF instrument using *nor*-hormane matrix in negative ion mode [45].

### 2.9. Attenuated Total Reflectance Infrared Imaging 

ATR-IR spectra were recorded on a Bruker Platinum Alpha A220/D01 spectrometer using the OPUS 6.5 software. Sixty four scans were averaged at a resolution of 4 cm^−1^ for the wavelength range from 500–4000 cm^−1^. Graphical processing was accomplished using the Microsoft Excel 2007 software package. 

### 2.10. Specific Surface Area and Average Fibril Diameter 

The Brunauer–Emmett–Teller specific surface area (SSA) of the NA was determined by nitrogen sorption at 77 K using a Micromeritics ASAP 2405 instrument [46]. The NA (0.033 g) was degassed in the Micrometrics ASAP 2405 at 100 °C for 4 h prior to the analysis, followed by N_2_ adsorption at 77.4 K.

The average fibril diameter (*d*) of NA was calculated from the BET specific surface area (Equation (7) [47], assuming a cylindrical shape of the cellulose fibrils and a skeletal cellulose density (ρ_SK_) of 1.46 g/m^−3^.

*d* = 4 (ρ_SK_/S_BET_) (7)

### 2.11. Porosity and Average Pore Size 

The porosity of the transducer NA was calculated using Equation (8) as the quotient of the transducer NA apparent density (Equation (9)) and cellulose skeletal density (1.46 g/cm^3^) [48]. The average pore size of NA was calculated from a 40-point nitrogen adsorption isotherm utilizing the MicroActive interactive data analysis software for TriStar II Plus 2.02 instrument (Micromeritics, Norcross, GA, USA) and a ramp rate of 10 °C/min over 240 min [47].

Porosity = (1 − ρ*_B_*/ρ*_SK_*) (8)

(9) $$\rho_{B}=\frac{m}{V}$$

### 2.12. Microscopy Studies of NAs 

Scanning electron microscopy (SEM) was used to study the morphology of the NA samples performed using a Tecnai Inspect S50 instrument (FEI, Hillsboro, WA, USA) under high vacuum at an acceleration voltage of 5.00 kV. All images were acquired with a magnification of 1000× (100 μm scale). The NAs were sputter coated with a 6-nm layer of gold using a Leica EM SCD005 instrument (Lecia, Buffalo, NY, USA). The Louisiana State University Shared Instrumentation Facility performed the field emission scanning electron microscopy (FE-SEM). The NA was also imaged using an FEI Quanta 3D FEG FIB/SEM instrument (FEI, Hillsboro, WA, USA) at a magnification of 65,000× (scale: 500 nm). The NAs was sputter coated with a thin 3-nm layer of gold-palladium using a Leica EM ACE600 instrument (Lecia, Buffalo Grove, IL, USA). A thin layer of coating was used to minimize the alteration of the surface morphology.

### 2.13. Fluorescence Response Assay

Preparation and characterization of the PepNA materials is outlined in previous work [49,50]. Phosphate buffer solution (PBS) (pH 7.4, 0.1 M sodium dihydrogen phosphate (NaH_2_PO_4_) and 0.5 M sodium chloride (NaCl) in Millipore water, filtered through a 0.2-μm filter) of the fluorescent peptide substrate was prepared in serial dilutions. A standard curve of HNE activity was constructed from the substrate solutions (1 down to 0.0156 μmol/mL). One well containing only PBS was included in the standard curve.

Each of the cellulosic and nanocellulosic matrices was cut (hole punched) to weigh ~2 mg per sample and placed into a 96-well plate. The PBS (100 μL) was added to each biosensor. The cotton cellulose nanocrystal (cCNC) stock solutions were prepared by suspending 20 mg of the cCNC in 1 mL of PBS (equivalent to 2 mg of the sample). To start the reaction, 50 μL of human neutrophil elastase (0.5 U/mL) were added to the wells containing the standard curve and to the wells containing the biosensors to provide a total volume of 150 μL. 

Fluorescent measurements at 37 °C were monitored for 1 hour at 1-minute intervals. The 96-well plate was shaken before each measurement for 3 s. The fluorescent measurements were acquired at 360-nm excitation and 460-nm emission wavelengths, respectively.

### 2.14. Molecular Modeling Studies

Computational studies were performed to calculate the minimization energy of the tripeptide (Suc-Ala-Pro-Ala-AMC) anchored to the cellulose model compound cellotriose [51]. The peptide substrate models were built using GaussView 5.0.9 (Gaussian Inc., Wallingford, CT, USA) and optimized using a semiempirical Hamiltonian method [Stewart 89, Steward89a] (PM3) contained in the Gaussian 09 Revision A.02 molecular orbital software [52,53].

## 3. Results and Discussion

### 3.1. Synthesis of Nanocellulosic Aerogels

**Figure 1:**
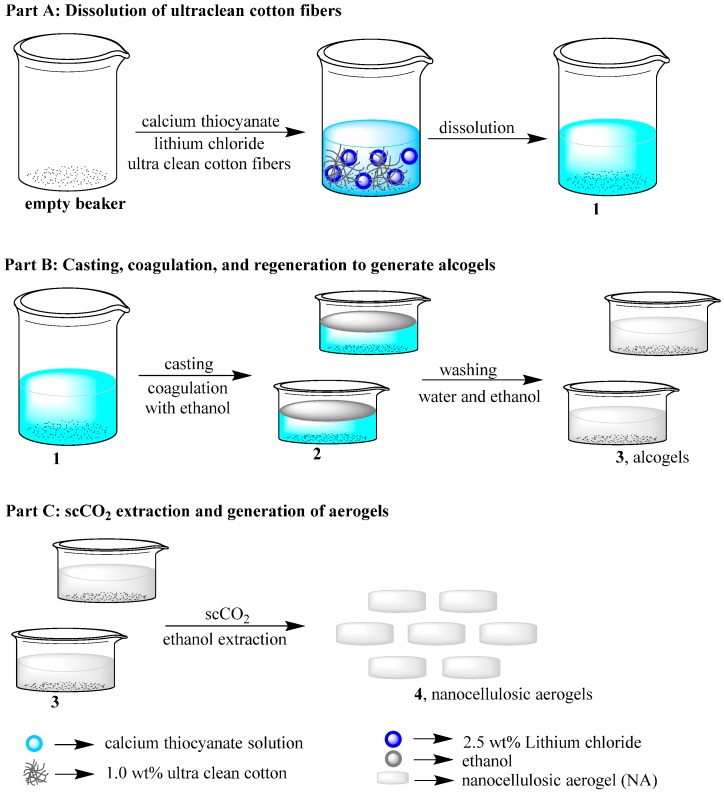
Schematic representation of the preparation of cellulose II aerogels.

The preparation of aerogels from greige cotton fibers was accomplished using the direct cellulose solvent calcium thiocyanate octahydrate. This salt hydrate has previously been utilized for the preparation of cellulosic aerogels [54,55]. Small quantities of lithium chloride (2.5 wt%) were added to facilitate cellulose dissolution [56]. Figure 1 illustrates the preparation of the nanocellulosic aerogels (NA) in all phases of dissolution, casting/coagulation, regeneration and supercritical carbon dioxide (scCO_2_) drying. Dissolution affords a homogenous solution of the raw material as confirmed by light microscopy. Scanning electron microscopy (SEM; see the nanocellulosic aerogel morphology section) revealed significant ballooning of the fibers during dissolution. It is also notable that a small quantity of undissolved fragments is visible via SEM, and these are thought to be due to cotton fiber cuticle and primary cell wall components, i.e., lipids and pectin (see the nanocellulosic aerogel morphology section), as discussed below. The dissolution procedure yields a molecular-dispersion when performed at a dilution percentage of 1% of cotton fibers. The use of calcium thiocyanate prevents shrinkage of the NA during the regeneration phase and provides good dimensional stability [55,56]. The preparation of the NA yielded a porous structure, which was characterized for properties conducive to a sensor transducer surface.

### 3.2. Synthesis of the Biosensor: Peptide-Nanocellulose Aerogel Conjugate

**Figure 2:**
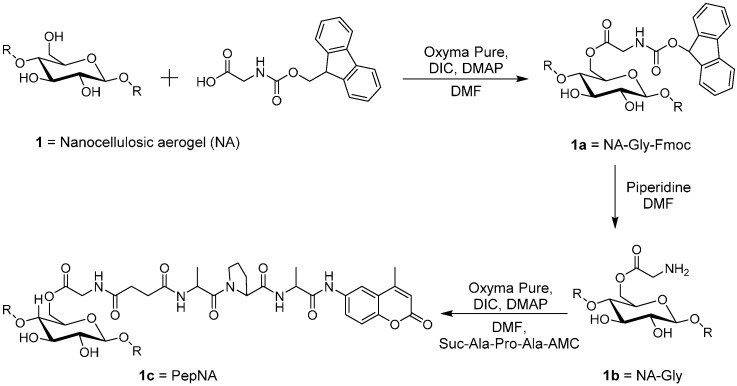
Scheme of cellulose esterification with glycine followed by tripeptide (Suc-Ala-Pro-Ala-4-amino-7-methyl-coumarin (AMC)) conjugation to the nanocellulosic aerogel. Note NA and PepNA are defined as nanocellulosic aerogel and peptide nanocellulosic aerogel, respectively. DIC, diisopropylcarbodiimide.

The peptide-based aerogel sensors of this study were designed based on previously-reported motifs that include a tripeptide substrate attached to glycine-esterified cellulose through a succinimidyl linker [57] (Figure 2). 

**Figure 3:**
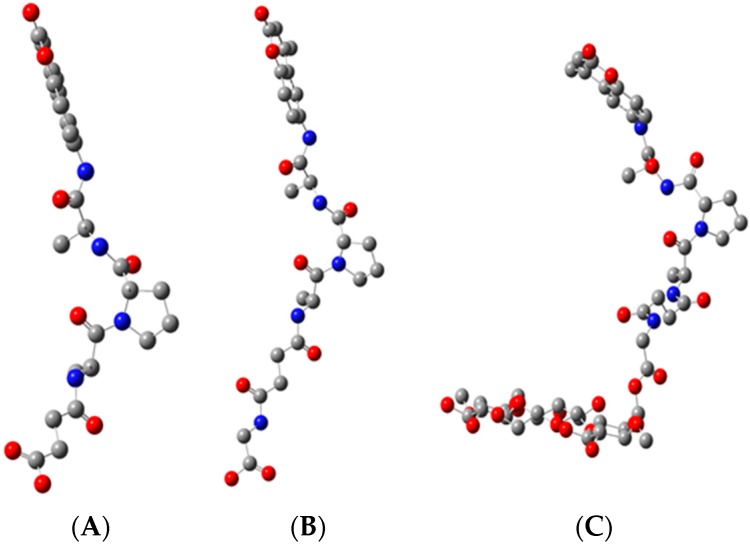
Minimum energy conformations of the tripeptide (Suc-Ala-Pro-Ala-AMC) (**A**) tripeptide coupled to glycine (**B**) and the tripeptide anchored onto glycinated cellotriose (**C**).

A molecular model of the peptide-nanocellulose aerogel (PepNA) conjugate prepared in this study is shown in Figure 3. Depicted is the tripeptide anchored to glycine esterified cellotriose, which would represent PepNA and contrasted with the free peptide analog at equilibrium (Figure 3). The disposition of the molecular models suggests some conformational differences in the sensor peptide conjugate that may result from interactions with the surface of the nanofibrillar cellulosic aerogel versus the peptide analog in solution. Previously, tripeptide sequences, such as Ala-Pro-Ala, have been described as inducing a β-turn conformation [58]. Thus, this feature combined with the nanofibrillar cellulosic surface may contribute to the higher affinity of HNE observed when compared with the same free untethered peptide analog. 

### 3.3. Characterization of the Peptide Nanocellulosic Aerogel

Table 1 lists the characterization profile of the peptide-nanocellulose aerogel (PepNA). The glycine residue is esterified to the NA resulting in a degree of substitution (D.S.) of 0.051 (Table 1). The incorporation of the peptide Suc-Ala-Pro-Ala-AMC (Pep) was at a level of 20 μg/mg and a D.S. of 0.015 and was confirmed by mass spectrometry giving the expected parent ion ([M + H] = 572.374). 

**Figure 4:**
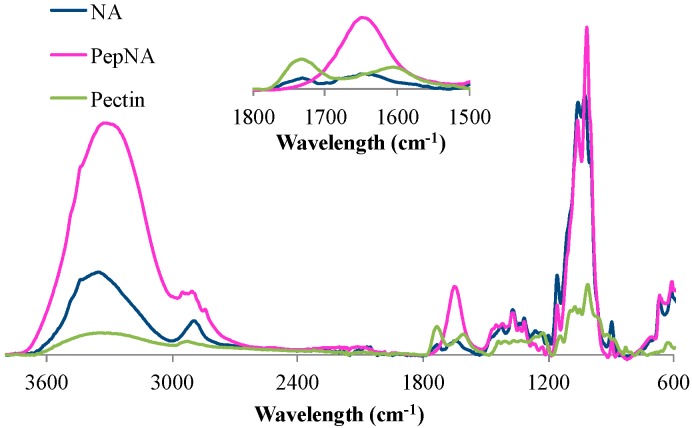
ATR-IR spectra of NA (blue), PepNA (pink) and pectin (green).

The IR spectra of the peptide-nanocellulosic aerogels shown in Figure 4 (bands listed in Table 2) are indicative of intramolecular hydrogen bonding and N-H stretching bands at 3480 cm^−1^ and 3435 cm^−1^ [59,60,61]. Furthermore, the bands at 3480 cm^−1^ and 3435 cm^−1^ may represent nanocellulose systems with a higher density of hydroxyl groups [50,59] compared to previously-reported cotton filter paper or print cloth [50]. The spectrum shows the amide peaks assigned to the tripeptide conjugate at 1652–1650 cm^−1^ (strong, literature value 1650) and 1568 cm^−1^ (weak, literature value 1540) [62,63,64]. The 1732 cm^−1^ band is indicative of C=O stretching vibrations (esters, aldehydes, e.g.), which is absent in the PepNA conjugate. This prompted the evaluation of commercially available pectin using ATR-IR, since pectin is composed of an α-(1 → 4)-linked D-galacturonic acid unit that varies in the ratios of carboxylic acids, methyl esters and O-acetyl esters within the pectin structure [65]. Comparison of the spectra revealed an overlapping peak at 1734 cm^−1^ [66,67] and indicates the presence of greige cotton-based pectin remaining in the NA. The ATR-IR results also support the ESI-MS findings that the tripeptide is anchored on the surface of the biosensor, and the presence of pectin carried through the preparative steps of the NA.

### 3.4. Structural Properties of Nanocellulosic and Peptide Nanocellulosic Aerogel

Table 3 lists the structural properties of the NA including SSA, average pore diameter, average fibril diameter, porosity and skeletal density. Nitrogen adsorption experiments at 77 K revealed a relatively high specific surface area of 163 m^2^·g^−1^, which is in good agreement with Pircher et al. [68], who obtained SSA of 190 m^2^·g^−1^ for aerogels prepared from 1.5 wt% solutions of cotton linters in the same solvent system. The average fibril diameter within the structure of the NA is calculated based on the BET surface area [47] and is estimated to be 17 nm. Pore size by gas absorption-mediated nanopore assessment of the NAs revealed an average pore diameter of 11 nm, which is consistent with a mesoporous and interconnected open porous structure that is usually found in cellulose II aerogels [68,69]. The porosity (99%) and density 12 mg/cm^3^, were calculated based on Equations (8) and (9), respectively. The SSA and porosity properties of the NA reported here are similar to the properties reported by Pircher et al. who prepared aerogels from cotton linters by (i) molecular-dispersing dissolution, (ii) coagulation of the biopolymer from the solution-state using ethanol as the anti-solvent and (iii) extraction of the organic solvent from the interconnected pore system using scCO_2_ [68]. Furthermore, Pircher et al. also evaluate the effect of different cellulose solvent systems including the calcium thiocyanate octahydrate and lithium chloride used herein on the morphological features at supramolecular and nano-structural levels and on bulk properties, such as strength or stiffness. Thus, although greige cotton and cotton linters vary somewhat in composition, preparation of aerogels from both cotton sources with the same cellulose solvent system gives rise to similar physical properties. 

The physical properties of the NA are similar for different sources of material when the method of preparation in terms of cellulose solvent, anti-solvent used for coagulation, solvent exchange protocol and drying method is the same. However, several correlations that affect the SSA are observable between the two sources of cotton with different cellulose solvent systems according to Pircher et al. Lower skeletal densities (1.529–1.689 g/m^3^) correlate to a higher SSA (328–163 m^2^·g^−1^). Furthermore, lower porosities (95%–99%) of the aerogels, greige cotton fibers and cotton linters with the different cellulose systems coincide with lower skeletal densities (1.529 g/m^3^–1.689 g/m^3^), respectively. 

Nanocellulose-based materials with a high SSA also have a high abundance of primary hydroxyls on the surface, which enables covalent attachment of the sensor molecules to the NA surface [50]. The NA of this study has an SSA of 163 m^2^·g^−1^ and D.S. levels of 0.051 and 0.015 for glycine esterification and peptide conjugation, respectively. Previous studies on derivatized cellulosic materials show that higher SSA values correlate with increased D.S. levels. For instance, cotton cellulose nanocrystals with an SSA of 186 m^2^·g^−1^ had a D.S. of 0.044 [28], which is three-fold higher than the D.S. for the NA. It is apparent that structural differences between nanocrystalline cellulose I and nanocellulose II forming the respective aerogels also influence the D.S. levels. Cellulose I nanocrystals typically have a cylindrical whisker shape resulting in high surface relative to crystal size dimensions of 159 nm in length and 15 nm in average fibril diameter [28]. On the other hand, nanocellulose II aerogels consist of 4–5 nm-thick fibrils that either form three-dimensional networks or aggregate to small spherical particles that agglomerate to open-porous networks, as well, depending on the phase separation mechanisms for the used cellulose solvent/anti-solvent system [68]. 

The lower peptide substitution of the NA may also occur due to solvent and surface chemistry effects during the peptide coupling, the accessibility of the hydroxyls on the highly porous surface and peptide orientation restrictions [50,57]. It is noteworthy that (i) double coupling of the glycine amino acid can counteract competitive reactions with pectins, (ii) potentially increase the D.S. levels of glycine esterification and (iii) peptide substitution on the NAs structure. 

### 3.5. Nanocellulose Aerogel Morphology

**Figure 5:**
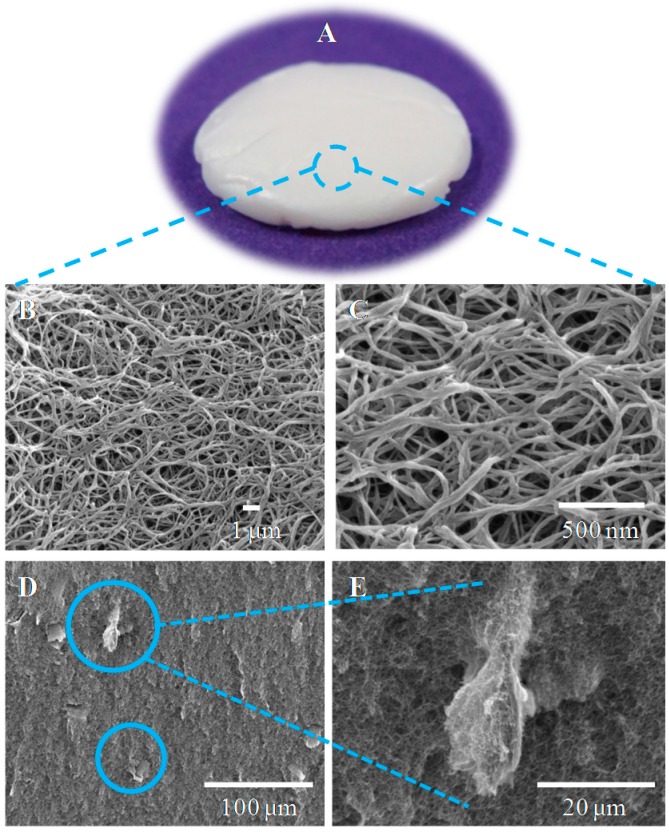
Microscopy images of NA: (**A**) optical image, (**B**) SEM image at 10,000× with 1 μm scale, (**C**) FE-SEM image at 80,000× with a 500-nm scale, (**D**) SEM image at 1000× with a 100-μm scale, (**E**) SEM image at 5000× with a 20-μm scale. The blue circles in (**D**) indicate undissolved fiber residues as magnified in (**E**).

The microscopy images from an optical lens scanning electron microscope (SEM) and field emission electron microscope (FE-SEM) are shown in Figure 5. Figure 5A shows the three-dimensional structure of the NA, and Figure 5B,C reveals the NA morphology dominated by an isotropic open porous, nanofibrillar network of coagulated cellulose II, which is a characteristic structural result of the cellulose solvent system employed (calcium thiocyanate octahydrate/lithium chloride) [54,68]. The FE-SEM images of the NAs also reveal the presence of mesopores (2–50 nm) and macropores (≥50 nm), which have an average diameter of 40 and 193 nm, respectively (Figure 5B,C). On the other hand, the BET SSA analysis of the NA indicated an average pore diameter of 11 nm, which is in agreement with a predominantly mesoporous pattern. It has been previously noted that a combination of techniques is required to identify the “true” range of pore sizes present in NA structures due to the fragility of these lightweight scaffolds [69] whose interconnected nanofibrillar architecture can be readily observed at the 500-nm scale. 

Figure 5D,E depicts the homogeneity of the network, which is only occasionally disturbed by discontinuities resulting from what is thought to be undissolved pectin closely integrated in the coagulated cellulose matrix. Round et al. extracted pectic polysaccharides from tomato plant cell walls with cycylohexane-trans-1,2-diaminetetraacetate (CDTA) and imaged the pectins with atomic force microscopy (AFM) [70,71]. The SEM images (Figure 5) show an elongated bulb-like structure that is closely similar to the AFM images previously reported. Although different sources of pectin, raw greige cotton or tomato plant cell walls and microscopy techniques were used, a similar bulb-like structure is consistent with pectin’s presence in the NA. 

**Figure 6:**
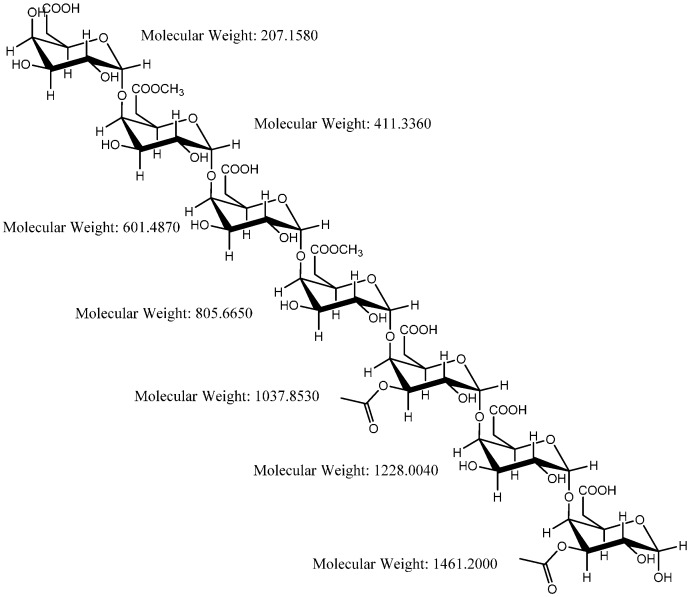
Proposed structure of pectin.

**Figure 7:**
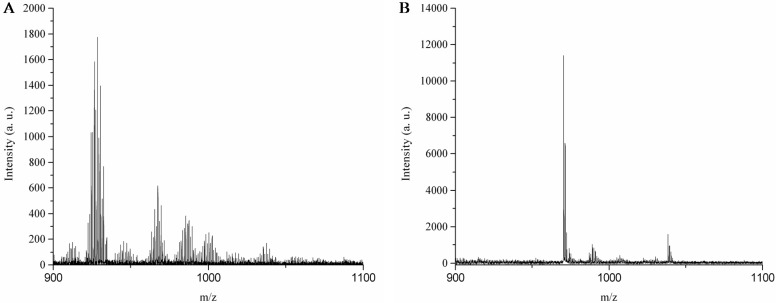
MALDI-MS spectrum of (**A**) commercially available pectin and (**B**) pectin extracted from NAs.

Pectin is a polysaccharide with a linear chain of α-(1 → 4)-linked D-galacturonic acid units, which vary in the proportions of methyl esters and O-acetyl esters [65]. The proposed structure of the pectins [72,73] present in the NAs is shown in Figure 6. To further identify the presence of pectin, the NA was extracted with hexane, and matrix-assisted laser desorption ionization time of flight mass spectrometry (MALDI-TOF-MS) was used to determine the molecular weight of the extracted pectin and commercially available pectin. A common *m*/*z* is present at [M]^+^ 1037.406 for the commercially available pectin and the extracted pectin from the NA (Figure 7).

It is noteworthy that the carboxylic acid groups of pectin may react during the peptide activation reaction with *N,N’*-diisopropylcarbodiimide (DIC) to produce an O-acylisourea [74,75]. This may account for the absence of the 1732 cm^−1^ band in the IR of the peptide nanocellulosic aerogel conjugate. 

### 3.6. Bioactivity Studies

The biosensing activity of the peptide-nanocellulose aerogel conjugate was determined by monitoring the reaction between the serine proteases human neutrophil elastase (HNE) and Suc-Ala-Pro-Ala-AMC peptide-nanocellulose aerogel conjugate (PepNA). The protease-catalyzed release of the COOH-terminal amino methyl coumarin fluorophore yields a fluorescence signal intensity that is indicative of the response and sensitivity of the PepNA conjugate biosensor to HNE. 

**Figure 8:**
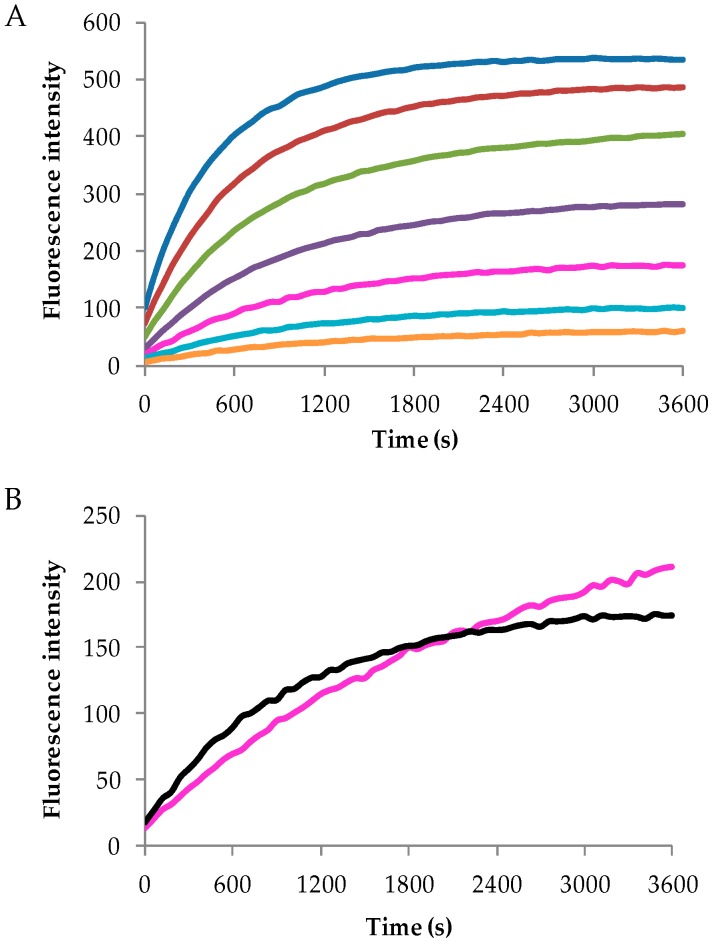
The fluorescence progress curves of the unbound or conjugated elastase substrate upon detecting the release of 7-amino-4-methylcoumarin triggered through serine protease human neutrophil elastase (HNE) at 0.5 U/mL (37 °C). (**A**) Calibrated curves of the unbound elastase substrate at 1 μM (blue), 0.5 μM (red), 0.25 μM (green), 0.13 μM (purple), 0.06 μM (pink), 0.3 μM (turquoise) and 0.015 μM. (**B**) The response curves of the unbound elastase peptide substrate (standard 0.06 μM, black) and 2 mg of the PepNA biosensor (pink).

Figure 8 shows the progress curves of the reaction of (A) unbound peptide substrate in solution (1–0.015 μM) and (B) the unbound peptide substrate in solution as a standard (0.06 μM) and 2 mg of the PepNA biosensor in a wound-like fluid with an HNE protease concentration of 0.5 units/milliliter (U/mL) (Table 4). As expected, a higher concentration (1 μM) of the unbound peptide substrate in solution reacts faster than a lower concentration (0.015 μM). The unbound peptidesubstrate (Suc-Ala-Pro-Ala-AMC) has a faster response with HNE, but the fluorescence intensity plateaus, whereas the NA with the bound peptide substrate has a slower response with a greater fluorescence intensity. The porous nature of the NA accounts for the delay in response considering that HNE must get to the surface and react with the substrate or penetrate a favorable pore size to react with the substrate, thereby reducing the reaction rate between the peptide substrate and HNE. On the other hand, a previous study by Edwards et al. investigated the progress curves of cotton nanocrystals substituted with the same tripeptide, which resulted in a greater fluorescence intensity [57]. As mentioned earlier, the nano-morphologies of nanocellulose II aerogels and cellulose I nanocrystals differ greatly with the nanocrystals, providing the maximum interaction with the large HNE enzyme. As observed from SEM and nitrogen adsorption studies, a wide range of nano-size pores (meso and macropores) is present throughout the NA, which may reduce the amount of peptide conjugated within the structure and inhibit the HNE enzyme from penetrating the structure due to its size, thereby resulting in a lower response and sensitivity. 

Table 4 lists the calculated response and sensitivity concentration of the NA. The calculated response concentration of 10.11 μmol/g indicates the amount of peptide substrate on 2 mg (0.002 g) of the PepNA biosensor reacting with the wound-like fluid containing HNE. Assessment of the sensitivity concentration or limit of detection required varying the concentration (2, 1, 0.5, 0.25, 0.13, 0.06 U/mL) of the wound-like fluid with 2 mg of PepNA. The PepNA effectively detected HNE in the wound-like fluid with a sensitivity of 0.13 U/mL. This is promising considering the PepNA was able to detect HNE at concentrations present in chronic wound fluid. Elevated levels of HNE vary from 0.02–0.1 U/mL depending on the type of chronic wound (diabetic, venous pressure and arterial ulcers) [33]. Although the PepNA is able to detect HNE levels for arterial ulcers, this is a start to creating a nanocellulosic-based biosensor as a point-of-care diagnostic capable of detecting HNE with selectivity and specificity.

### 3.7. Application of PepNA Sensors

The three-dimensional open porous structure is an ideal function for a semi-occlusive wound dressing, since it enables gas exchange between the wound bed and environment [76], cell permeation [77,78] and prevents wound dehydration [76]. The presence of pectin and the source of cellulose in the NA creates an absorbent structure since other commercially available hydrocolloid dressings incorporate gelatin and pectin to increase absorbency [79,80]. Another beneficial aspect of the aerogel is its biosensor function with high SSA that enables a higher loading of the elastase peptide on the porous nanofibrillar systems, which improves sensor sensitivity functions. Furthermore, the NA may behave as a potential sequestrant of the cationic serine protease, HNE, present in the wound fluid due to its negative surface charge that derives from cellulose [81,82].

The use of the PepNA biosensing element incorporates intelligent features by the use of sensor functions, such as color, pH and temperature change, by activating or driving cellular responses [83] that can signify when a dressing needs replacing or, as proposed herein, as a way to detect HNE proteases present in wound exudate. The combination of intelligent and semi-occlusive features, due to the porous features of the NA, in a single dressing is not available at this time; however, such a system would be capable of providing point-of-care diagnostics for the prevention of chronic wounds by detecting proteins that dominate the wound exudate. Therefore, interfacing the PepNA biosensor into a multilayered dressing offers a way to gage the effectiveness of treatment while providing an effective and robust way to monitor protease levels in chronic wounds.

## 4. Conclusions

Low density nanocellulosic II aerogels have been successfully prepared from greige cotton by (a) dissolution in calcium thiocyanate octahydrate, which contained 2.5 wt% of lithium chloride; (b) casting; (c) cellulose coagulation and washing; (d) solvent exchange and (e) supercritical carbon dioxide drying, which yield an open porous and interconnected structure. 

The conjugation of the elastase tripeptide (Suc-Ala-Pro-Ala-AMC) to the aerogel was successful using peptide chemistry. Characterization of the aerogel indicated a highly porous structure with varying pore sizes and a high SSA structure with wetting and absorbent properties. The morphology of the derived aerogels is dominated by the fibrous cellulose II network whose homogeneity is occasionally disrupted by small quantities of undissolved residues, which however seem to be well integrated into the aerogel matrix. 

The aerogel biosensor detects HNE at concentrations found in chronic wounds and has a four-fold greater affinity for HNE versus the free peptide substrate. Although PepNA has a limited sensitivity concentration of 0.13 U/mL and is able to detect HNE at levels found in arterial chronic wound fluid (0.1 U/mL), expanding its sensitivity concentration (<0.1 U/mL) to target all chronic wound types (diabetic and venous pressure) would be ideal. The PepNA biosensors when compared with other nanocellulosic sensors demonstrates the need for improving reactivity with HNE for its potential to function as a component of multilayered intelligent protease sequestrant dressings. In spite of the bioactivity of the NA, it remains a beneficial transducer surface as a biosensor layer for an intelligent protease sequestrant wound dressing.

## Figures and Tables

**Table 1:** Mass spectral, quantitative peptide incorporation and degree of substitution values obtained for the aerogels.

Name ^a^	ESI-LC-MS ^b^ (m/z)	Average N (%)	Peptide (μg/mg)	D.S. ^c^
Aerogel-Gly		0.43		0.051
PepNA	[M + H] 572.374	0.76	20.31	0.015

^a^ The abbreviation Pep indicates the peptide conjugated with glycine (Gly-Suc-Ala-Pro-Ala-AMC). ^b^ The [M] calculated for C27H33N5O9 is *m*/*z* 571.23 and includes the glycidyl link attached to the peptide. ^c^ D.S. is the degree of substitution as calculated by the Touzinsky and Gordon method.

**Table 2:** Characteristic ATR-IR bands for the NA, PepNA and pectin.

Name	Wavenumber (cm^−1^)	Literature (cm^−1^)	Assignment
NA	3496 and 3443	3486–3439	Intramolecular hydrogen bonding
	3352	3570–3200	ν(O―H) stretching vibration
	2895	3000–2800	ν(C―H, C―CH_3_, C―H_2_) stretching
	1732	1762	ν(C=O) stretching of COOH
	1635	1633	ν(H_2_O) absorbed molecules
	1427	1429	ν(C―H) wagging-in-plane bending
	1370–1315	1372, 1336	ν(C―H) bending, ν(O―H) in-plane
	1262	1204, 1320	bending, and ν(C―H) wagging
	1024	1042	ν(C―O) stretching vibration
	896	898	ν(C―H_2_ and C―OH) deformations and
			ν(C―O―C) stretching at β glucosidic
	668	700–600	ν(C―C) stretching vibration
PepNA	3480 and 3435	3486–3439	Intramolecular hydrogen bonding
	3328	3570–3200	ν(N―H) stretching vibration and ν(O―H) stretching vibration
	2950–2842	3000–2800	ν(C―H, C―CH_3_, C―H_2_) stretching
	1649	1650	ν(N―H) amide I (stretching) and amide II
Pectin	3339	3570–3200	ν(O―H) stretching vibration
	2926	3000–2800	ν(C―H, C―CH_3_, C―H_2_) stretching
	2655	2700–2500	ν(O―H) stretching vibration of COOH
	1734	1740–1705	ν(C=O) stretching of COOH

**Table 3:** Specific surface area (SSA), density, thickness, porosity, average pore and fibril diameter values obtained for the aerogel.

Name	SSA ^a^ (m^2^·g^−1^)	Skeletal Density (g/cm^3^)	Thickness (μm)	Porosity (%)	Average Pore Diameter (nm)	Average Fibril Diameter (nm)
Aerogel	162.943	1.689	383	98.8	11	16.8

^a^ The specific surface area, porosity, average pore size and average fibril diameter of the aerogel were calculated using nitrogen adsorption with the Brunauer–Emmet–Teller theory.

**Table 4:** The response calculated concentration and sensitivity concentration of the peptide aerogel conjugate.

Name	Response Calculated Concentration ^a^ (μmol/g of Biosensor)	Sensitivity Concentration ^b^ (U/mL)
PepNA	10.11	0.13

^a^ The calculated response of the peptide aerogel conjugate (2 mg) upon detection of 7-amino-4-methylcoumarin released with HNE at 0.5 U/mL substrate hydrolysis at 37 °C. ^b^ The sensitivity concentration of the peptide aerogel conjugate (2 mg) upon detection of 7-amino-4-methylcoumarin released with HNE at 2, 1, 0.5, 0.25 and 0.125 U/mL substrate hydrolysis at 37 °C.
